# The decline in dental caries among Korean children aged 8 and 12 years from 2000 to 2012 focusing SiC Index and DMFT

**DOI:** 10.1186/s12903-016-0188-x

**Published:** 2016-03-22

**Authors:** Han-Na Kim, Dong-Hun Han, Eun-Joo Jun, Se-Yeon Kim, Seung-Hwa Jeong, Jin-Bom Kim

**Affiliations:** Department of Dental Hygiene, College of Health Sciences, Cheongju University, Cheongju, Korea; Department of Preventive and Social Dentistry, School of Dentistry, Seoul National University, Seoul, Korea; Department of Preventive and Community Dentistry, Pusan National University, School of Dentistry, 49, Busandaehak-ro, Mulgeum-eup, Yangsan-si, Gyeongsangnam-do, 626-870 Seoul, Korea; BK Plus Project of Center of Center for Professional Education on Technosciences of Oral Health, Seoul, Korea

**Keywords:** Children, Dental caries, Decayed, missing, and filled teeth, Significant caries index

## Abstract

**Background:**

The aim of this study was to analyse the prevalence and severity of dental caries among Korean children aged 8 and 12 years over a period of 12 years by determining the number of decayed, missing, and filled teeth (DMFT) and the Significant Caries index (SiC index).

**Methods:**

Stratified cluster-sampled data from the National Oral Health Survey conducted from 2000 to 2012 were analysed. In 2000, 2006, and 2012, a total of 2397, 2650, and 9601 children aged 8 and 12 years were examined, respectively. The children’s oral health status, including the number of DMFT and fissures sealed teeth, was examined and recorded. The SiC index was calculated according to the child’s residential district.

**Results:**

Over the 12-year period, the percentages of caries-free children aged 8 and 12 years increased from 26.0 to 42.7 % and from 53.4 to 69.6 %, respectively. The percentages of children aged 8 and 12 years with sealed teeth in 2012 were 62.1 and 62.5 %, respectively, more than triple the rates in 2000. The mean DMFT values of children aged 8 and 12 years decreased from1.04 to 0.67 and from 2.86 to 1.84, respectively. The SiC index of children aged 8 and 12 years also decreased from 2.73 to 1.97 and from 6.13 to 4.51, respectively. The rate of reduction in DMFT among 8- and 12-year-old children in the second 6 years of the observation period was lower than that in the first 6 years.

**Conclusions:**

A remarkable decline in dental caries of 8- and 12-year-old Korean children was observed over the 12-year study period. The mean DMFT values and SiC index of children aged 8 and 12 years decreased. The reduction rate between 2000 and 2006 was higher than that between 2006 and 2012.

## Background

Dental caries can have a negative impact on children's quality of life [1]. Sheiham [2] reported that severe dental caries affect young children's growth and hypothesised that improving dietary intake and treating dental caries in pre-school-aged children would increase growth rates and improve quality of life. To monitor dental caries in children, the World Health Organization (WHO) developed an oral disease surveillance system. The first global map with data related to decayed, missing, and filled teeth (DMFT) for 12-year-olds was presented in 1969 [3]. There has been an overall decline in the prevalence and severity of dental caries in children and adolescents around the world, particularly in developed countries [4]. In spite of this overall decline, scores or indices expressing the prevalence of dental caries have varied. A previous study reported differences in the prevalence of dental caries in permanent teeth between 2000 and 2010. The numbers of DMFT in 12-year-olds were 2.96, 2.07, 0.89, and 0.70 in the Czech Republic, Brazil, Denmark, and Germany, respectively [5]. Some studies have demonstrated inequalities in the distribution of dental caries [6–8]. Socioeconomic factors, such as access to oral health care, can contribute to these inequalities [9].

The use of DMFT data is an accepted approach for assessing the severity and prevalence of dental caries as well as for assessing overall oral health [10]. However, skewed distributions of caries prevalence have been reported in many countries. Ditmyer et al. [11] reported that the mean number of DMFT did not accurately reflect the skewed distribution of dental caries in youth in Nevada, leading to incorrect conclusions. The Significant Caries index (SiC index) was proposed as a means to highlight individuals with the highest caries scores in each population [12, 13].

Since the Oral Health Act for oral health promotion was passed by the National Assembly of the Republic of Korea in 2000, public oral health programmes have been developed to reduce the incidence of dental caries. In Korea, there is a legal basis for supporting oral health programmes; the development of specific public oral health programmes by municipal authorities is required by the Oral Health Act. The Health Promotion Act states that health information and healthy environments should be provided to promote general health. The operation of public health centres and the installation of regional health centres were included in the Regional Health Act [14]. Public oral health programmes comprising community water fluoridation, fluoride mouth rinsing, and a fissure sealant program were the primary programmes for preventing dental caries in urban and rural areas [15]. Kim and Jeon [16] showed that the DMFT values in 12-year-olds had declined and that general oral health had improved but that its inequality had increased. Several studies using cross-sectional or other designs have reported data related to Korean children’s oral health using DMFT values [17, 18] and the SiC index [19] to express the inequality of oral health [16]. In this study, we used a simple index based on the number of DMFT, the SiC index, and the SiC index/DMFT ratio to express disparities in the incidence of dental caries. Because the SiC index/DMFT ratio is calculated as the SiC index divided by the DMFT score, it can be used as a novel expression of the severity of dental caries for groups based on region or community. For example, if the mean number of DMFT declines but the prevalence of severe dental caries based on the SiC index remains high, the SiC/DMFT ratio would increase. Thus, the SiC index/DMFT ratio can show disparities in the prevalence of dental caries for high-risk groups. The aim of the present study was to analyse the prevalence and severity of dental caries in 8- and 12-year-old Korean children over a period of 12 years by measuring the number of DMFT and determining the SiC index.

## Methods

### General characteristics related to oral health in Korea

The population increases between 2000 and 2006 was 2.7 %, and its increase between 2006 and 2012 was 3.5 %. More than 50 million people lived in Korea in 2012. The gross domestic product (GDP) increased in 2012 from that in 2006. Less than 10 % of the GDP total expenditures was spent on health. The number of practicing dentists in 2000 was 17,647. The number of practicing dentists in Korea increased by 28.5 % in 2006 compared to that in 2000 and by 18.2 % in 2012 compared to that in 2006. Since 1979, the Korean government has placed dentists in rural areas where the private dental care system was inferior. The percentage of the Korean population covered by the water fluoridation programme that began in 1981 [20] decreased from 12.7 to 6.1 % between 2000 and 2012. The decline was caused by a movement banning fluoridation since 1998. A fluoride mouth-rinsing programme was begun in 1983, and a free fissure sealant programme that was implemented by the government from 2002 to 2009 was conducted for almost 200,000 primary school children every year by public health centres. Fissure sealant has been covered for children by national health insurance since 2009. The scope of oral health in public health care has expanded since 2005. Facilities for tooth-brushing drills in primary schools were installed beginning in 2011 (Table 1). Because a previous study reported that more than 97 % of metropolitan citizens used fluoridated toothpaste [21], it was assumed that the majority of Korean people used fluoridated toothpaste and that the toothpaste had preventive effects against dental caries.

**Table 1:** General features of Korea and the characteristics of oral health services

	2000	2006	2012	%, Change ^b^	%, Change ^c^
Population (thousands persons)	47,008	48,297	50,004	2.7	3.5
0 to 14 years old	9,911	8,996	8,422	−9.2	−6.4
Health expenditure/GDP (%)	4.8	6.0	7.4^a^	25.0	23.3
Total expenditure on health (% GDP)					
Practicing dentists (persons)	14,410	18,515	21,888	28.5	18.2
Population drinking fluoridated water (%)	12.7	5.8	6.4	−54.3	10.3
Population received public fissure sealant program aged 12 year		34.0	62.54	-	83.9

^a^ Indicated data in 2011

^b^ Results from (2006–2000)/2000

^c^ Results from (2012–2006)/2006

### Subjects

The National Oral Health Survey has been conducted every 3 years by the Ministry of Health and Welfare based on the Oral Health Act legislated by the National Assembly of the Republic of Korea in 2000 [22, 23]. The aim of the survey is to collect representative oral health data for the establishment of public oral health policy [23]. To promote the oral health of all Korean people, enforced by the regulations of the Oral Health Act, a survey of the population’s oral health status should be conducted by the Ministry of Health and Welfare. Based on the Oral Health Act, ethics committee approval was not needed. Written consents were obtained from participants and their parents. The data of national oral health survey can be obtained from the Korea Health Promotion Foundation [24] by sending an e-mail with a written application. Children aged 8 and 12 years were included in the surveys in 2000, 2006, and 2012.

### Sampling

Children’s oral health statuses varied based on socioeconomic status (SES) and living environment, and living standards could vary according to the size of counties, cities, and metropolitan areas. Therefore, a population size was classified by county, city, and metropolitan area.

Samples were selected using a stratified cluster-sampling procedure based on a population and housing census in order to represent all Korean children. The surveyed areas also included counties, cities, and metropolitan areas. The distribution of primary schools was determined by the results of the sample allocation. After allocation, one school located in each surveyed district was selected as a representative school. Consequently, the numbers of selected schools were 200, 150, and 190 in 2000, 2006, and 2012, respectively. The National Oral Health Survey for schoolchildren was conducted through school visits.

The sampling frame for the 2000 survey was the list of schools from the 200 survey districts maintained by the Department of Statistics. Children and adolescents aged 6 to 17 years from primary, junior, and high schools in the 200 surveyed districts were selected randomly from a list according to the surveyed districts. The total number of subjects was 11 947, and the numbers of subjects aged 8 and 12 years were 1194 and 1203, respectively. After one classroom of each school’s grade was randomly selected, every fifth child in the selected classroom was examined by stratified clustering [25].

The sampling frame for the 2006 survey was the same as that for the 2000 survey, except for the number of surveyed districts and subjects in the focus age group. In the 2006 survey, 2- to 16-year-old children and adolescents (10 649 subjects) were included in the oral survey. Among the total number of subjects, the numbers of 8- and 12-year-old children were 875 and 1775, respectively. The number of surveyed districts changed to 150, and the number of sampled 12-year-olds was intentionally twice as high as that of the other age groups [26].

The number of surveyed districts changed to 190 and subjects comprised 5-, 8-, 12-, and 15-year-old children in the 2012 survey. The sampling frame for the 2012 survey was the same as that for the 2000 and 2006 survey. The proportional distribution based on the total population scale was applied to allocate 190 districts according to each city and prefecture, then to sub-grouping, and it was also applied to each city and prefecture. Each kindergarten, primary, junior, and high school in the 190 survey districts was selected randomly from a list dividing all the schools according to the survey districts [27]. In Korea, almost all primary schools are operated by the government; thus, there were few socio-economic differences in the educational conditions among sampled schools.

### Oral examination and data collection

The survey was conducted with the cooperation of the Department of Education in each survey district. Prior to the survey, principals of selected schools received a letter requesting their cooperation, and questionnaires were sent out for completion and parents’ agreement.

Fifteen survey teams composed of university staff majoring in preventive dentistry in dental schools and dental hygiene schools joined in the 2000 survey. All oral status examiners were dentists who worked as professors or assistants in universities. For the 2006 survey, eight teams composed of public health dentists and university staff majoring in preventive dentistry in dental schools and dental hygiene schools joined the survey. For the 2012 survey, 10 teams were composed of two public health dentists and eight university staff members from dental schools. Each team in the 2000, 2006, and 2012 survey contained a dentist and an interview expert [28].

The oral examinations were performed in the classroom, with children sitting facing a window with a portable blue-white colour spectrum examination light. The diagnostic criteria followed the WHO protocol [29]. Dental caries were recorded as D_3_ caries into the dentine threshold. Teeth showing sealed fissures were identified as being either complete or incomplete. Examinations were performed using disposable instruments: plane mirrors, sharp explorers, and ball-ended Community Periodontal Index probes. Radiographs were not used for diagnosis.

To control the quality of the national oral health examination, calibration training was conducted before each survey. After the principal investigator and examiners performed oral examinations on the same subjects, they discussed any different findings. An examiner was accepted to the survey when inter-examiner variability was acceptable and the findings showed good agreement with those of the principal investigator (kappa ≥0.7).

### Statistical analysis

The mean number of DMFT and the prevalence of caries in permanent teeth were calculated based on the clinical examination during the 2000, 2006, and 2012 surveys. The prevalence of caries-free (DMFT = 0) and fissures sealed (individuals with pit and fissure sealants) was also calculated. The SiC index was calculated as the mean DMFT value for the one-third of the population with the highest caries scores.

The mean number of DMFT number and the SiC index were used as dependent variables. Age and area of residence were included as independent variables. To measure any inequality related to children’s oral health, the SiC index/DMFT ratio was used [28]. A univariate analysis was performed to determine frequencies and mean values. The level of significance was set at *p* < 0.05.

## Results

Table 2 shows the number of subjects, percentage of caries-free children, and percentage of children with sealed teeth. In 2000, 2006, and 2012, a total of 2397, 2650, and 9601 children aged 8 and 12 years were examined, respectively.

**Table 2:** Prevalence of caries experience and fissure sealant in Korea 2000–2012 by age

Year	Living region	Age 8					Age 12				
		N	DMFT=0, %	P*	Sealed, %	P*	N	DMFT=0, %	P*	Sealed, %	P*
2000	Total	1194	53.4	0.67	19.2	0.877	1203	26.0	0.006	14.8	0.011
	Urban	780	52.9		19.4		784	28.6		16.7	
	Rural	414	54.3		18.8		419	21.2		11.2	
2006	Total	875	69.5	1.00	38.6	<0.001	1775	39.1	0.041	33.0	0.237
	Urban	694	69.5		35.3		1386	40.4		33.8	
	Rural	181	69.6		51.4		369	34.1		30.4	
2012	Total	4379	69.6	0.559	62.1	0.367	5222	42.7	0.612	62.5	0.438
	Urban	3781	69.2		62.3		4565	42.8		63.0	
	Rural	598	76.3		58.3		657	41.0		55.4	
	P-value**										
			DMFT=0, %		Sealed, %			DMFT=0, %		Sealed, %	
2000 - 2006	Total		<0.001		<0.001		Total	<0.001		<0.001	
	Urban		<0.001		<0.001		Urban	<0.001		<0.001	
	Rural		<0.001		<0.001		Rural	<0.001		<0.001	
2006 - 2012	Total		0.034		<0.001		Total	<0.001		<0.001	
	Urban		0.08		<0.001		Urban	0.044		<0.001	
	Rural		0.158		<0.001		Rural	<0.001		<0.001	

*p-value for chi-square test

**p-value for t-test

The percentages of caries-free children aged 8 years increased from 53.4 to 69.6 % between 2000 and 2012 (*p* < 0.05). The percentage of caries-free children aged 8 years was the highest in rural areas in 2012 (76.3 %). Overall, the percentages of caries-free children aged 8 years were lower in urban areas than in rural areas, but not significantly so. The percentage of 8-year-old children with sealed teeth increased from 19.2 to 62.1 % from 2000 to 2012 (*p* < 0.001). The percentage of 8-year-old children with sealed teeth in rural area (51.4 %) was higher than that in urban area (35.3 %) in 2006 (*p* < 0.001).

Over the 12-year study period, the percentage of caries-free 12-year-old children increased from 26.0 to 42.7 %. For the same age group, the percentage of children with sealed teeth in 2012 was 62.5 %. Compared to that in 2000, it had increased fourfold. The percentage of caries-free children was higher in urban areas than in rural areas in all three surveys. The average annual increases in the percentages of caries-free children from 2000 to 2006 were 12–13 %, while the increases in the subsequent six years were lower, at 3–6 %.

Table 3 shows the DMFT and SiC index results. Over time, both values decreased for all ages. The mean number of DMFT for children aged 8 years in 2000 (1.04) was significantly lower than that in 2006 (0.68; *p* < 0.001), while the mean DMFT index in 2012 (0.67) decreased slightly compared to the mean in 2006 (0.68; *p* = 0.207). Between 2000 and 2006, the reduction rate of the number of DMFT (50.55 %) was higher in rural areas among children aged 8 years than in urban areas. The mean SiC index of 8-year-old children also decreased significantly (from 2.73 to 2.02) between 2000 and 2006. However, the mean SiC index seemed to be similar between 2006 and 2012, and there was no statistically significant difference.

**Table 3:** Change of the mean DMFT and SiC indices of Korea 2000-2012 by age

Age		2000			2006			2012^a^			Reduction rate^b^, %		Reduction rate^c^, %	
		DMFT	SiC	SiC/DMFT	DMFT	SiC	SiC/DMFT	DMFT	SiC	SiC/DMFT	DMFT	SiC	DMFT	SiC
8	Total	1.04	2.73	2.63	0.68	2.02	2.97	0.67	1.97	2.94	**−35.58**	**−27.84**	−1.47	**−2.48**
	Urban	1.12	2.93	2.62	0.69	2.08	3.01	0.68	2.00	2.94	**−39.29**	**−31.74**	−1.45	**−3.85**
	Rural	0.91	2.36	2.59	0.61	1.85	3.03	0.45	1.43	3.18	**−50.55**	**−39.41**	−26.23	**−22.70**
P-value*		**0.015**	0.544		0.437	**<0.001**		0.162	0.823					
12	Total	2.86	6.13	2.14	2.16	5.16	2.39	1.84	4.51	2.45	**−35.66**	**−26.43**	**−14.81**	**−12.60**
	Urban	2.77	6.06	2.19	2.09	5.06	2.42	1.82	4.48	2.46	**−34.30**	**−26.07**	**−12.92**	**−11.46**
	Rural	3.02	6.26	2.07	2.40	5.57	2.32	2.09	4.92	2.35	**−30.79**	**−21.41**	**−12.92**	−11.67
P-value*		0.144	0.676		**0.045**	0.314		0.159	0.205					

Statistically significant values bolded (*p*<0.05)

^a^ Values were calculated considering a complex sample design

^b^ Results from (2012–2000)/2000

^c^ Results from (2012–2006)/2006

*p-value for t-test

Twelve-year-old children showed the same trends. The mean DMFT index of children aged 12 years consistently decreased, from 2.86 to 2.16 and finally to 1.84, over the 12-year period (*p* < 0.05). The reduction rates of the mean DMFT index among 12-year-old children were 12.9 to 35.6 % compared to the past 6 years (*p* < 0.05). Similar decreases were found for the SiC indices of 12-year-old children. The SiC indices of 12-year-old children decreased from 6.13 to 4.51 (*p* < 0.05) during the 12-year period. However, the reduction rate between 2000 and 2006 was higher than that between 2006 and 2012.

**Fig. 1:**
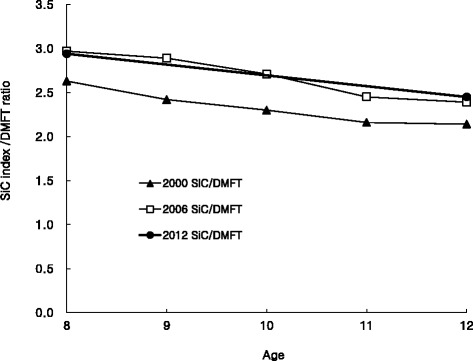
The relationship expressed by SiC index over DMFT in 2000-2012

Figure 1 shows that the SiC index/DMFT ratios of children aged 12 years in 2006 and 2012 were higher than those in 2000. The SiC index/DMFT ratios in 2006 and 2012 were similar for children 12 years.

**Fig. 2:**
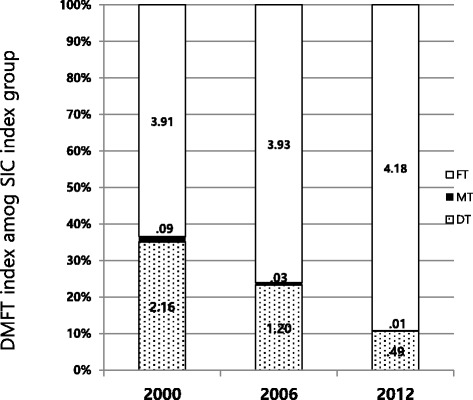
Mean SiC index in 12-year-old children in Korea, 2000-2012

Figure 2 shows the DMFT scores across the different ranges of SiC index values and the number of each decayed, missing, and filled teeth in children aged 12 years. As the DMFT scores decreased from 6.16 to 4.68, the number of missing and decayed teeth decreased while the number of filled teeth increased, from 3.91 to 4.18.

## Discussion

The results of this study show that the prevalence and severity of dental caries in Korean 8- and 12-year-old children declined between 2000 and 2012, according to national oral health data collected from a large nationally representative sample.

The percentage of caries-free children aged 12 years increased from 26.0 to 42.7 % over 12 years, and the majority of this increase occurred in the first 6 years. These findings are in agreement with the results of Lee and Han [30], who reported that the prevalence of caries decreased from 75.9 to 60.5 % in 12-year-olds between 2003 and 2010. Park et al. [31] demonstrated that the improvement in children’s oral health was attributable to the public fissure sealant programme in Korea as well as to improvements in living standards and health behaviour during these first 6 years. The relatively lower increase in the number of caries-free children between 2006 and 2012 may be related to slow economic growth [32] and the fact that the Department of Oral Health in the Ministry of Health and Welfare was established in 1997 but abolished in 2007. The abolishment of the Department of Oral Health in the Ministry of Health and Welfare indicates that there had not been additional public health programmes developed to improve children’s oral health. Eaton et al. [33] reported that dental hygienists were more frequently employed in some countries (Denmark, Finland, Iceland, the Netherlands, Norway, and Sweden) where dentists or clinicians worked as chief dental officers compared to their employment in other countries where chief officers in charge of oral health were not dentists or clinicians. It is therefore likely that the Department of Oral Health in the Ministry of Health and Welfare in Korea had played an important role in public oral health.

The percentage of children with fissure-sealed teeth continuously increased between 2000 and 2012. Since the public fissure sealant programme started with the support of the government budget in 2002, more than half of 8-year-old children living in rural areas had sealed teeth in 2006, and the difference in 8-year-old children with sealed teeth between urban and rural areas was 16.1 % (*p* < 0.001). In rural areas, all children underwent fissure sealant operations without charges; meanwhile, in urban areas, economically disadvantaged children could benefit from the public fissure sealant programme in public health centres without charges. The gaps between the urban and rural areas may have been related to the number of dental hygienists working at public health centres. Dental hygienists have been placed in public health centres located in rural areas since 1986, so they could work for school oral healthcare and public oral educational programmes. After that, the fissure sealant programme was covered by National Health Insurance for children aged 6–14 years in December 2009, but the public free-of-charge fissure sealant programme ceased in 2010; this could have led to the smaller increase in the number of children with sealed teeth in rural, comparatively unprivileged areas. Edelstein and Chinn [34] demonstrated that disparities in dental visits continued to be evidenced by age, family income, race/ethnicity, and caregiver education. Children whose parents did not have enough time to bring children to dental clinics could not benefit from fissure sealant covered by National Health Insurance.

The mean DMFT values of children aged 12 years continuously declined, from 2.86 to 1.84, over 12 years, which was the first time that the mean DMFT of children aged 12 years was lower than 2.0 since the National Oral Health Survey started in 2000. Cho et al. [35] reported that the mean DMFT scores for 11-year-old children in fluoridated and non-fluoridated areas in 2011 were 1.05 and 1.83, respectively. Despite this decline, the DMFT values of children aged 12 years remains higher than those in other countries. Bernabe and Sheiham [5] reported the DMFT numbers of 12-year-olds based on published reports from 26 countries. Among these countries, the DMFT numbers of 12-year-olds in the UK, Finland, Denmark, and Canada were 1.10, 0.07, 0.89, and 1.02, respectively.

Because the SiC index takes into account the DMFT scores, the SiC indices also declined with the decrease in mean DMFT scores. The decline gradient for the SiC index was higher than the decline gradient for DMFT scores. Nishi et al. [13] reported that if the total mean DMFT in the whole group decreased by a certain amount, half of the decrease was due to a change in the sub-group used to calculate the SiC index. The increase in the number of caries-free children and the decrease in SiC index may have contributed to the overall decline in mean DMFT; however, these do not fully explain the decrease in mean DMFT. In other words, the SiC index/DMFT ratio represents the difference between the SiC index and the DMFT scores of 12-year-olds. This ratio increased in 2006 compared to that in 2000. However, the ratio in 2012 was similar to that in 2006 and higher than that in 2000. Differences in severity of dental caries still existed, and the gap was worse between 2006 and 2012 than that between 2000 and 2006.

This study confirmed the gradual decline in dental caries among 8- and 12-year-old children between 2000 and 2012. Fluoridated toothpaste was speculated as a potential factor contributing to the decline in dental caries among Korean children. It has been known as the most important reason for the decline in dental caries in developed countries between the 1970s and 1980s [36]. In Korea, almost 97 % of people used fluoridated toothpaste [21]. Lee and Han [30] also demonstrated that the potential determinant of the decline in the number of caries was fluoridated toothpaste. Another use of fluoride for caries prevention was the water fluoridation programme operated in some regions in Korea. Kim et al. [37] assessed the effects of the fluoridation programme on dental caries. The caries preventive fraction was estimated by assessing the differences in DMFT scores. The numbers of DMFT for 12-year-old children in areas with a fluoridation programme and control areas were 1.60 and 2.12, respectively, with an estimated prevention effect of 24.7 %.

The main finding of this study was the change in DMFT scores among the sub-group used to calculate the SiC indices (Fig. 2). The numbers of missing and decayed teeth declined, whereas the number of filled teeth increased between 2000 and 2006. The increased number of filled teeth was the primary factor contributing to the slower decline in DMFT scores. More attention should be given to the fact that coverage of light-curing composite-resin restoration by National Health Insurance in 2018 could influence the number of filled teeth. Jung et al. [19] showed that a significant proportion of the sub-group used to calculate the SiC index had no sealed fissures on the first molar and lived in rural areas. The public oral health programme to provide fissure sealant by the government budget could be reinforced to reduce the gaps between rural and urban areas. Previous studies reported that significant oral health disparities persist [16, 38, 39]. Patrick et al. [39] reported that children living in poverty are likely to have limited access to dental care, and the improved Medicaid programmes with acceptable reimbursement rates may encourage dentists to treat them.

This study has several limitations, including that we could not consider detailed socioeconomic factors. In the National Oral Health Survey, questionnaires about the parents’ socioeconomic factors were collected separately from the oral health data in 2000. Truin et al. [40] reported that the decline in caries in 12-year-olds of low SES has come to an end. However, in medium- and high-SES groups, the percentages of caries-free children have continued to increase, suggesting that the trend in the prevalence of caries could be affected by SES levels. Consequently, socioeconomic factors need to be considered in future studies. Oral health is often associated with individual-level determinants, such as oral hygiene and dietary habits. Further research is warranted in order to determine ways to reduce inequalities in oral health among children.

## Conclusions

Although this study lacked information on children’s SES and detailed data on individual oral health and diet conditions, a remarkable decline in dental caries for 8- and 12-year-old children was observed during the 12-year study period. Public oral health programmes using fluoride and fissure sealants and the common use of fluoridated toothpaste may have contributed to the improved oral health of 8- and 12-year-old Korean children. To continue oral health promotion, strategies that support current programmes and coverage for populations with severe dental caries should be considered.

## Abbreviations

DMFT: decayed, missing, and filled teeth
SiC index: Significant Caries index
WHO: World Health Organization
GDP: gross domestic product
SES: socioeconomic status
